# On the design of EEG-based movement decoders for completely paralyzed stroke patients

**DOI:** 10.1186/s12984-018-0438-z

**Published:** 2018-11-20

**Authors:** Martin Spüler, Eduardo López-Larraz, Ander Ramos-Murguialday

**Affiliations:** 1 Department of Computer Engineering, Wilhelm-Schickard-Institute, University of Tübingen, Sand 14, 72076 Tübingen, Germany; 2 Institute of Medical Psychology and Behavioral Neurobiology, University of Tübingen, Silcherstr. 5, 72076 Tübingen, Germany; 3 TECNALIA, Health Technologies, Neural Enginering Laboratory, Mikeletegi Pasalekua 1, 20009 San Sebastian, Spain

**Keywords:** Neuroprostheses, Brain machine interface (BMI), Rehabilitation robotics, Proprioceptive feedback, motor rehabilitation, stroke, Neurotechnology

## Abstract

**Background:**

Brain machine interface (BMI) technology has demonstrated its efficacy for rehabilitation of paralyzed chronic stroke patients. The critical component in BMI-training consists of the associative connection (contingency) between the intention and the feedback provided. However, the relationship between the BMI design and its performance in stroke patients is still an open question.

**Methods:**

In this study we compare different methodologies to design a BMI for rehabilitation and evaluate their effects on movement intention decoding performance. We analyze the data of 37 chronic stroke patients who underwent 4 weeks of BMI intervention with different types of association between their brain activity and the proprioceptive feedback. We simulate the pseudo-online performance that a BMI would have under different conditions, varying: (1) the cortical source of activity (i.e., ipsilesional, contralesional, bihemispheric), (2) the type of spatial filter applied, (3) the EEG frequency band, (4) the type of classifier; and also evaluated the use of residual EMG activity to decode the movement intentions.

**Results:**

We observed a significant influence of the different BMI designs on the obtained performances. Our results revealed that using bihemispheric beta activity with a common average reference and an adaptive support vector machine led to the best classification results. Furthermore, the decoding results based on brain activity were significantly higher than those based on muscle activity.

**Conclusions:**

This paper underscores the relevance of the different parameters used to decode movement, using EEG in severely paralyzed stroke patients. We demonstrated significant differences in performance for the different designs, which supports further research that should elucidate if those approaches leading to higher accuracies also induce higher motor recovery in paralyzed stroke patients.

## Background

Brain machine interfaces (BMI) have been applied to motor rehabilitation in stroke patients with promising results [1–7]. A recent work from our group demonstrated, in a double-blind controlled study, that BMI training combined with behavioral physiotherapy can elicit significant and relevant motor functional recovery [2]. One of the main hypothesis of our previous work was related to the use of ipsilesional activity for BMI, since it has been demonstrated that, after stroke, patients present bilateral or enhanced contralesional brain activity during motor tasks, and only patients shifting brain activation towards ipsilesional areas presented motor recovery in the acute phase [8, 9]. Therefore, the BMI feedback, contingent with the ipsilesional motor cortex activity, was postulated as the key factor required for the functional improvements.

Despite the main organizational principle of primate motor systems is that cortical areas control the movements of the contralateral limbs, these areas also play a role in ipsilateral movements [10–12]. In fact, it has been shown how BMIs using primates’ intracranial ipsilateral activity could perform as well as BMIs using contralateral activity [12, 13], or perilesional activity in rodents [14]. In humans with stroke, when bihemispheric electroencephalographic (EEG) activity is considered to decode movement commands (i.e., motor attempt or motor imagery) of the paralyzed limbs, the achieved performances are higher than if only ipsilesional activity is considered [15, 16]. Nevertheless, it is unclear if using a BMI to link contralesional or bihemispheric brain activity with proprioceptive feedback in the paretic limb would elicit the same or better functional results than if ipsilesional activity is used [2] and it is out of the scope of this manuscript. However, as BMI can create a causal link between EEG oscillatory activity and behavior (i.e. between brain and movement), BMI control represents motor control and, if improved, motor learning. In this manuscript we discuss different option to create that link (BMI design) to optimize motor control (BMI performance).

To date, different BMI designs have been proposed to decode movement commands in severely or completely paralyzed stroke patients. However, the relationship between the BMI design—and its performance—and potential for motor functional recovery is still an open question. In the decoding of movement information from EEG data, there are several factors that can significantly influence the performance. One of them is the type of classifier used. Linear classifiers are generally preferred in rehabilitative BMI setups (despite non-linear ones can often provide higher performances [17]). The main reason is their simplicity, since a linear threshold should be enough to efficiently detect some of the EEG correlates of movement intentions: e.g., the event-related (de)synchronization (ERD/ERS) of sensorimotor rhythms (SMR). The use of adaptive classifiers that update their parameters over time has been proposed as a way to deal with EEG non-stationarities, which can be advantageous for multi-session interventions [18], although the improvements in performance using these approaches have not been quantified in real scenarios with stroke patients. Although the type of classifier used can influence the final result, the way the features are extracted, selected and processed is the most influential step [19]. This can involve the movement correlate selected and the brain area from which it is recorded, but also the way that the signals are processed before being analyzed by the classifier.

Generally, BMI parameters for rehabilitation applications are selected based on prior knowledge or a priori hypotheses [17, 18, 20, 21]. Recent trends have also proposed the use of residual electromyographic (EMG) activity combined with EEG to build hybrid BMI systems [22, 23], since some patients, even with severe paralysis, show decodable EMG patterns [24]. Evaluating the role of the BMI design on functional recovery requires studying the effects in different groups of patients, and the number of patients required grows exponentially with the number of parameter combinations, making it an effort difficult to afford. However, the BMI designs that provide highest performances could be determined offline, and therefore it may allow re-designing future interventions based on the parameters that offer a better control of the rehabilitation robot. Higher BMI performances have been correlated with larger excitability enhancement in healthy subjects [25] and motor recovery in stroke patients [7].In the here presented work, we propose a systematic analysis of different configurations for a non-invasive BMI to decode motor intentions in severely paralyzed stroke patients, quantifying their effects on performance. We make use of the dataset recorded in our previous study, which involves 32 patients during 4 weeks of daily BMI training [2], and we expanded it with 5 more patients, making it the largest dataset of its kind. We simulated offline the performance that the BMI would have obtained under different conditions, varying: (1) the cortical source of activity (i.e., ipsilesional, contralesional, bihemispheric); (2) the type of spatial filter applied; (3) the EEG frequency band; (4) the type of classifier. In addition, we evaluated also motion intention decoding using residual EMG activity of the paretic muscles.

## Methods

The present study aimed at evaluating the influence of different configurations for a BMI on the obtained decoding accuracy. Therefore, we ran different tests on a fixed dataset, varying several parameters offline, but simulating an online BMI in a rehabilitation scenario. The dataset analyzed in this work was recorded during our previous double-blind controlled clinical study [2], and we expanded it with 5 more patients that performed an additional control condition.

### Patients

Thirty-seven participants with chronic stroke (18 male; mean age 53.7 ± 11.9 years, range 29 to 73 years; interval since stroke 66.7 ± 59 months, range 10 to 232 months; Table 1) were recruited through the University Hospital of Tübingen. The recruitment criteria included: age between 18 and 80 years, complete paralysis of one hand without ability for active finger extension, interval since stroke of at least 8 months, no psychiatric or neurological condition other than stroke, no cerebellar lesion or bilateral motor deficit, no epilepsy, Mini-Mental State (MMS) score above 21 (for more details see [2]).

**Table 1:** Details of patients

Pat. #	Group	Gender	Age (years)	Months since stroke	Lesion side	cFMA
1	Exp.	M	69	72	L	5.5
2		M	51	139	R	24
3		F	35	60	R	25.5
4		M	48	45	R	7.5
5		M	70	23	L	8
6		M	57	122	R	17
7		M	29	25	R	15
8		M	60	130	L	9.5
9		F	35	28	R	11
10		F	53	30	L	5
11		F	36	16	L	11
12		F	72	44	L	2
13		F	55	45	L	16.5
14		M	65	45	R	3.5
15		M	47	80	R	12
16		F	52	156	L	5.5
17	Sham	F	73	23	R	1
18		M	51	16	L	3.5
19		M	50	215	L	33.5
20		F	55	17	R	0.5
21		M	54	121	R	16
22		F	66	23	L	16.5
23		F	54	10	L	8
24		M	69	89	R	26
25		M	40	53	R	3.5
26		M	47	232	R	13.5
27		M	66	48	R	7.5
28		M	58	28	R	8.5
29		M	40	46	L	30.5
30		F	53	20	L	17.5
31		M	63	120	L	8.5
32		M	55	51	L	22.5
33	C-	F	65	67	L	8.5
34		F	65	131	L	7.5
35		M	65	99	L	7
36		F	31	15	L	33.5
37		M	60	14	L	13
Avg.	16 Exp/16 Sham/5 C-	22 M/15 F	54.4 ± 11.9	67.5 ± 56.4	16 R/21 L	12.6 ± 8.9

Group indicates if the patient performed the Experimental—contingent positive condition (Exp), the sham condition, or the contingent negative condition (C-). Lesion side indicates the damaged brain hemisphere. cFMA stands for combined Fugl-Meyer assessment, which comprises hand and arm motor scores combined, excluding coordination, speed and reflexes (range 0–54 points, with 54 points indicating normal hand/arm function)

### Data acquisition

EEG data were acquired with an Acticap system (BrainProducts, GmbH, Munich, Germany) from 16 electrodes, placed at Fp1, Fp2, F3, Fz, F4, T7, C3, Cz, C4, T8, CP3, CP4, P3, Pz, P4 and Oz, with the ground in AFz and the reference in FCz (modified 10/20 system). Four EOG electrodes were used to record horizontal eye movements for both eyes and vertical eye movements for the right eye. Surface electromyographic (EMG) activity was recorded from both arms, using 8 bipolar Ag/AgCl electrodes from Myotronics-Noromed (Tukwila, WA, USA) and a bipolar amplifier from Brainproducts GmbH, Munich Germany. EMG was recorded on top of 4 different muscle groups (extensor carpi ulnaris, extensor digitorum, external head of the biceps and external head of the triceps) in order to detect the movement onset and involuntary muscle contractions. The Brainamp amplifiers and signal processing module were connected through a client-server architecture, with the amplifier acting as the server and the signal processing module running on a stand-alone client PC. Data were sampled at 500 Hz and transmitted over to the client PC for storage and real-time signal processing using the BCI2000 platform (www.bci2000.org) [26].

### BMI intervention

The intervention aimed at rehabilitating the paretic upper-limb of the patients and involved two phases. In the first phase, the patients trained their upper arm with an arm orthosis that allowed reaching movements, and in the second phase, they trained their ability to open and close the hand with a hand orthosis (see 2.4). Each patient changed from the first to the second phase when he/she was able to extend the arm correctly (with gravity compensation), or when the 8th training session was reached.

The patients were divided in one experimental group and two control groups, and all of them performed a daily BMI training for 4 weeks. All the subjects received the same instructions: to attempt to move their paralyzed arm/hand following audiovisual cues, so that the BMI would detect their movement intentions and control a robotic orthosis that would actually move their limb. In the experimental group (contingent positive feedback group), the decrease of SMR power over the ipsilesional motor cortex was linked to the movement of the orthosis. In the first control group (contingent negative feedback group), the increase of SMR power over the ipsilesional motor cortex controlled the orthosis. In the second control group (sham feedback), the movements of the orthosis were random and independent of the brain activity, although the amount of time the orthosis was moving was kept in the same range as in the experimental group (between 55 and 80% of each trial).

**Fig. 1:**
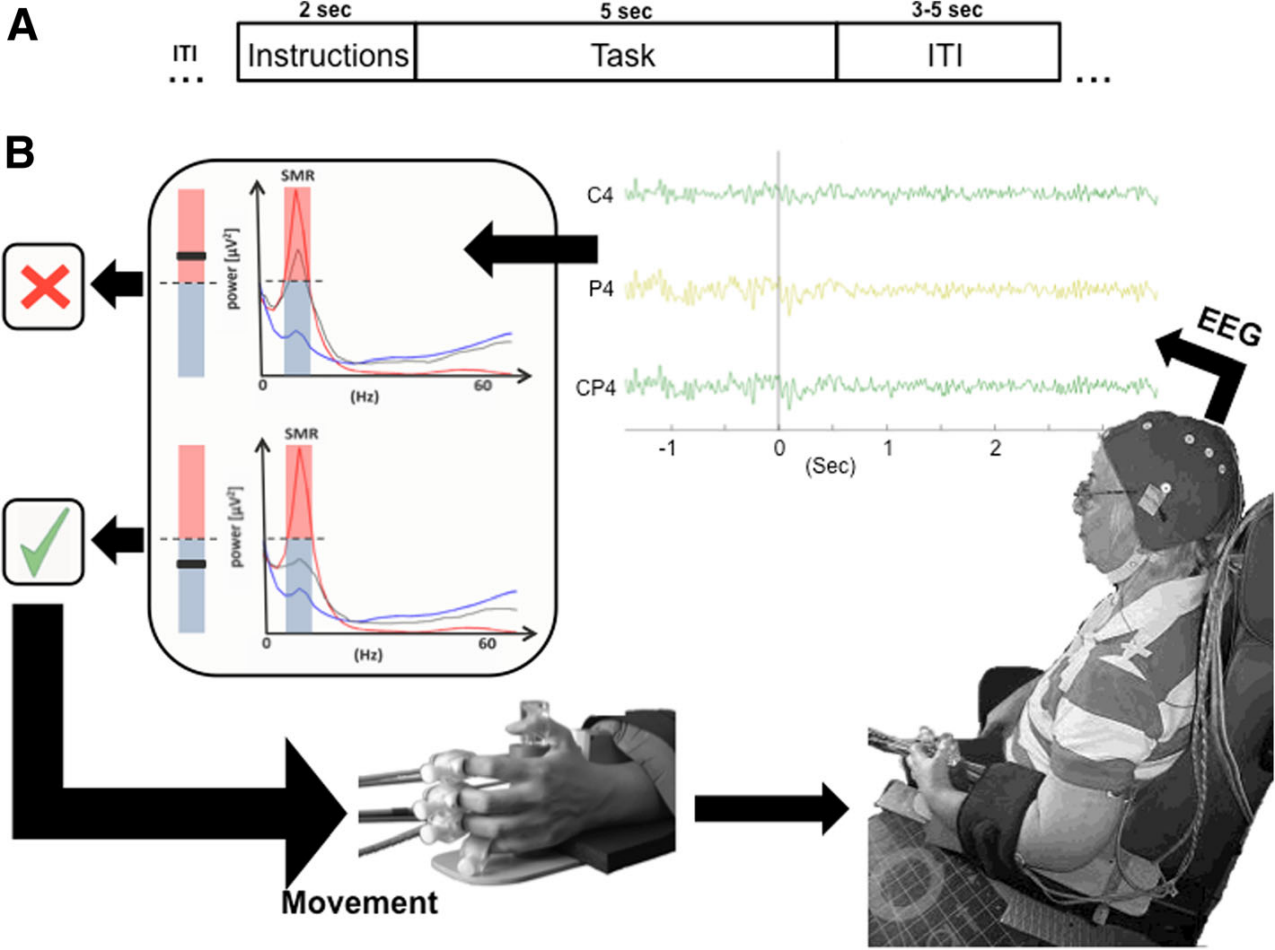
Timing and BMI functioning. **a**) Timing of each trial of the experiment. Each trial starts with an inter trial interval (ITI) of 3 s followed by an auditory instruction period for the task (“Try to move the Left/Right hand”). 2 s after the instruction, a start cue is presented and 5 s later an end cue is presented. **b**) The patient’s EEG from ipsilesional electrodes is processed online and transformed into power of the sensorimotor rhythm (SMR). The BMI generates 2 distributions of data, one for resting (red area: during ITI) and one for trying to move (blue area: during task). The classification threshold is the middle between the 2 distributions mean (dashed line between the red and grey shaded areas). When the power of SMR is 5 consecutive times on the same side of the threshold (classified 5 consecutive time as rest or trying to move) the orthosis will change its status (from stop to move or move to stop)

The day before the first training session the patients performed an EEG-screening (patients were asked to open and close the hand for 5 s at their own pace upon an audiovisual imperative cue that indicated whether the left, the right or none of the hands had to be moved, with a randomized inter-trial interval of 5 to 7 s), which was used to identify the individual SMR features (electrodes and frequency bins) to calibrate the BMI classifier. These features were selected by visual inspection as the electrode-frequency pairs that had highest R-square values [27] when comparing the brain activity of resting state versus attempting to open and close the paretic hand. The electrodes on the motor cortex in the ipsilesional hemisphere evaluated were C3/4, CP3/4, P3/4, as F3/4 were considered to be potentially corrupted with eye or forehead muscle activity and central line electrodes might contain too much contralateral activity due to the volume conduction effect (see Table 2). During the BMI intervention (4 weeks of daily training, excluding weekends) the patients were attached to a robotic exoskeleton and performed, in each session, between 15 and 20 blocks of 17 trials each. Auditory cues were used to instruct the patients during the trials: first a “warning” cue was presented and 2 s afterwards a “Go” cue initiated the trial, which lasted for 5 s (See Fig. 1). During this time, the orthosis was controlled according to the brain activity of the patient (in the experimental and first control groups) or randomly (in the second control group). The BCI2000 two-class classifier (motor intention versus rest) sent an output every 40 ms to the orthosis, requiring five consecutive classifier outputs of the same condition (i.e. detecting either intention to move or rest five consecutive times), to send the orthosis a no-move (zero velocity value) or a move (positive velocity) command. The SMR power was computed with an autoregressive (AR) model [28] over a sliding window of 500 msec. The BMI software maintained a history of the mean SMR power from each trial and assigned this to a distribution representing observations for the two classes (rest or motor intention). The classification threshold, defined as the mean distance to the two distributions, was adaptive to account for changes in the shapes of these distributions over the course of training.

**Table 2:** Electrode-frequency pairs used during the online intervention

Pat. #	Group	Channel	Frequency
1	Exp.	P7	11.5–14.5
2		C4, P4, P8	8.5–11.5
3		C4, P4, P8	8.5–11.5
4		C4, P4, P8	8.5–11.5
5		P7	5.5–8.5
6		C4, P4	14.5–17.5
7		C4, P8	17.5–20.5
8		C3	11.5–14.5
9		C4	5.5–8.5
10		C3, P3, P7	8.5–11.5
11		C3	5.5–8.5
12		C3	5.5–8.5
13		P7	5.5–8.5
14		C4, P4, P8	8.5–11.5
15		C4	5.5–8.5
16		P7	5.5–8.5
33	C-	F4, C4, P8	14.5–17.5
34		F4, C4, P8	17.5–20.5
35		F4, C4, P4, P8	20.5–23.5
36		C4, P4, P8	8.5–11.5
37		C4, P8	9.5–12.5

Group indicates if the patient performed the Experimental—contingent positive condition (Exp), or the contingent negative condition (C-). Patients from the sham group are excluded from this table since they did not receive closed-loop feedback. Channel and frequency correspond to the electrodes and frequencies used to provide contingent feedback during the intervention

### Orthoses

For the arm movements, the patients used a ReoGo rehabilitation robot (Motorika, Cesarea, Israel), acting as arm orthosis. With the arm of the patients attached to the robotic orthosis, we recorded a 3D movement imitating a reaching action (customized to each patient range of movement). This trajectory was used to provide kinesthetic feedback to the patients, who could control moving or stopping the orthosis along the pre-recorded trajectory.

The hand orthosis was an in-house made robotic device. Each finger was moved individually using a DC − Motor (M-28 from Kaehlig Antriebstechnik GmbH, Hannover, Germany) with worm gearhead for each finger. This motor drove via cogwheel and cograil a 5-m bowden cable. A finger holder was mounted on the other side of each bowden cable. Near this finger holder, an optical position sensor was mounted to detect the finger position independent of the bowden cable tolerance and elasticity to correct finger positioning. The power electronics were made as a linear regulation to prevent artifacts from switching devices to the EEG (more information can be found in [2, 29]). The BMI system sent the hand orthosis positioning and velocity commands, and the movement was determined by the difference between current and desired position.

### Offline analysis of EEG decoding

To evaluate how the choice of the electrode placement, spatial filtering, frequency band, and classifier influences the decoding accuracy, we performed an offline analysis (using MATLAB). To simulate an online scenario, we used a time window of 500 ms, shifted it in 40 ms steps (as done in the actual intervention) over the whole EEG signal and used the data in this window for classification. This offline analysis was done for each patient and each session separately.

#### Electrode placement

Regarding the electrodes used to compute the features, we compared three different spatial distributions:

1. **Ipsilesional side only**: C3, CP3, P3 for patients with lesion on the left hemisphere; and C4, CP4, P4 for patients with lesion on the right hemisphere.
2. **Contralesional side only**: C4, CP4, P4 for patients with lesion on the left hemisphere; and C3, CP3, P3 for patients with lesion on the right hemisphere.
3. **Both hemispheres**: C3, C4, CP3, CP4, P3, P4.

#### Spatial filter

Spatial filters are applied to EEG activity to obtain reference-free signals [30]. We compared three different configurations: (1) no spatial filter, (2) common average reference (CAR), or (3) a small Laplacian (*finite*-method from fieldtrip [31]).

#### Frequency band

The spectral features were estimated using a 16th-order autoregressive model computed with the Burg method [28] in the range of 1 to 40 Hz (resolution of 2 Hz per bin). After power spectrum estimation, the logarithm function was applied to each value. Then, we evaluated the influence on decoding accuracy of relying on: (1) the alpha band only (8–12 Hz), (2) the beta band only (15–30 Hz), or alpha and beta bands together (8–30 Hz),

#### Classifier

For classification, we compared the simple linear classifier used for online feedback during the BMI training [2, 29], and an adaptive approach based on support vector machines (SVM), as proposed in [32]. This comparison allows evaluating the relevance of adaptation of classifier parameters, which has been pointed as an important aspect to deal with EEG non-stationarities, especially in multi-session interventions [18]. The SVM classifier was implemented using the LibSVM toolbox (with a linear kernel and hyperparameter C = 1). To continuously adapt the classifier and adjust to possible non-stationaries in the EEG signal, the SVM was retrained every 400 ms. For training the SVM, 2 buffers with data were used with each storing the data (power spectra computed on 500 ms windows) of the last 2 min of rest period and the last 2 min of movement period. Every 400 ms new data was added to the corresponding buffer (each buffer depending on the period, either rest or movement) and the SVM was trained using the data from these two buffers. To guarantee online feasibility of this adaptive SVM method, the update method by [33] was used. Using this method, we do not have to completely train the SVM from scratch each time new data is added to the buffer, which would be very time consuming. Instead, the method allows to update the existing SVM solution and update it incrementally with each new data. In the same way, if old data is removed from the buffer, the SVM solution can be updated decrementally to consider the removal of old data from the buffer. Thereby, this adaptive SVM procedure is a computationally efficient way to have an SVM trained on the latest data in each step.

#### Metric

No additional signal processing and artifact removal was applied to the data to resemble online real-scenario conditions. To evaluate the decoding accuracy (DA), we calculated the difference between true positives (TP; percentage of movement classified during movement period) and false positives (FP; percentage of movement classified during rest period). A perfect classification would result in a TP-FP value of 1, while a value of 0 would results from random control. We relied on TP-FP because this metric is independent of the classifier threshold, and therefore can deal with coupled TP and FP biases that are caused by an offset in the classifier output (e.g., a classifier having high TP and high FP should have a similar result as one having low TP and low FP). This metric is equivalent to the accuracy obtained by averaging TP and true negatives (TN; which is computed as 1-FP). Thus, for BMI-based stroke rehabilitation, we try to maximize the number of TP, while minimizing the number of FP, to optimize the proprioceptive and visual feedback provided.

### Offline analysis of EMG decoding

The EMG signal was bandpass filtered between 5 Hz and 200 Hz (order 10 Butterworth filter). Then, the waveform length (WL) was calculated for each of the EMG channels [34], which is defined by:

$$ WL=\sum \limits_{k=1}^n\mid {x}_k-{x}_{k-1}\mid $$

Where *n* is the number of EMG data points of the window to analyze, *x*_*k*_ is the *k* data point in that window and *x*_*k-1*_ is the previous data point. To evaluate EMG activity as an alternative to the BMI, we implemented an equivalent movement decoder, with a window of 500 ms shifted in 40 ms steps. In every step, the WL from the EMG channels was used as input features for the classifier. Different combinations of EMG electrodes were used as input for the classifier. First, we evaluated the influence of extracting the EMG features from: (1) the paretic arm, (2) the healthy arm, or (3) both arms. In this case, all the 4 muscles were considered (i.e., 2 on the forearm extensors, one on biceps and one on the triceps). Then, we compared the performances when extracting the EMG features from: (1) only the muscles involved in the task (i.e., the 2 distal electrodes for hand opening/closing, or the 2 proximal electrodes for upper-arm movement), or (2) all 4 electrodes [35]. Additionally, we compared the two classifiers explained in the section above (simple linear classifier vs adaptive SVM).

### Statistical analysis

To investigate how the different designs influence the performance of the BMI, we performed several statistical comparisons. A linear regression model [36] was fitted to the data, explaining the motion intention decoding accuracy of each session based on the various factors, and we used an n-way ANOVA to test for significant influence of these factors on the decoding accuracy. Paired comparisons were conducted using a Wilcoxon’s rank-sum test with Bonferroni correction for multiple comparisons, if needed.

## Results

We separated the analyses in 2 sections for clarity. In the first section, we report the influence of each of the four studied parameters (electrode placement, spatial filter, frequency band, and classifier) on BMI performance, measured as TP-FP. In the second section, we compared EMG and EEG (muscle vs brain) movement intention decoding accuracy.

### Comparing parameters for optimal decoding accuracy

**Fig. 2:**
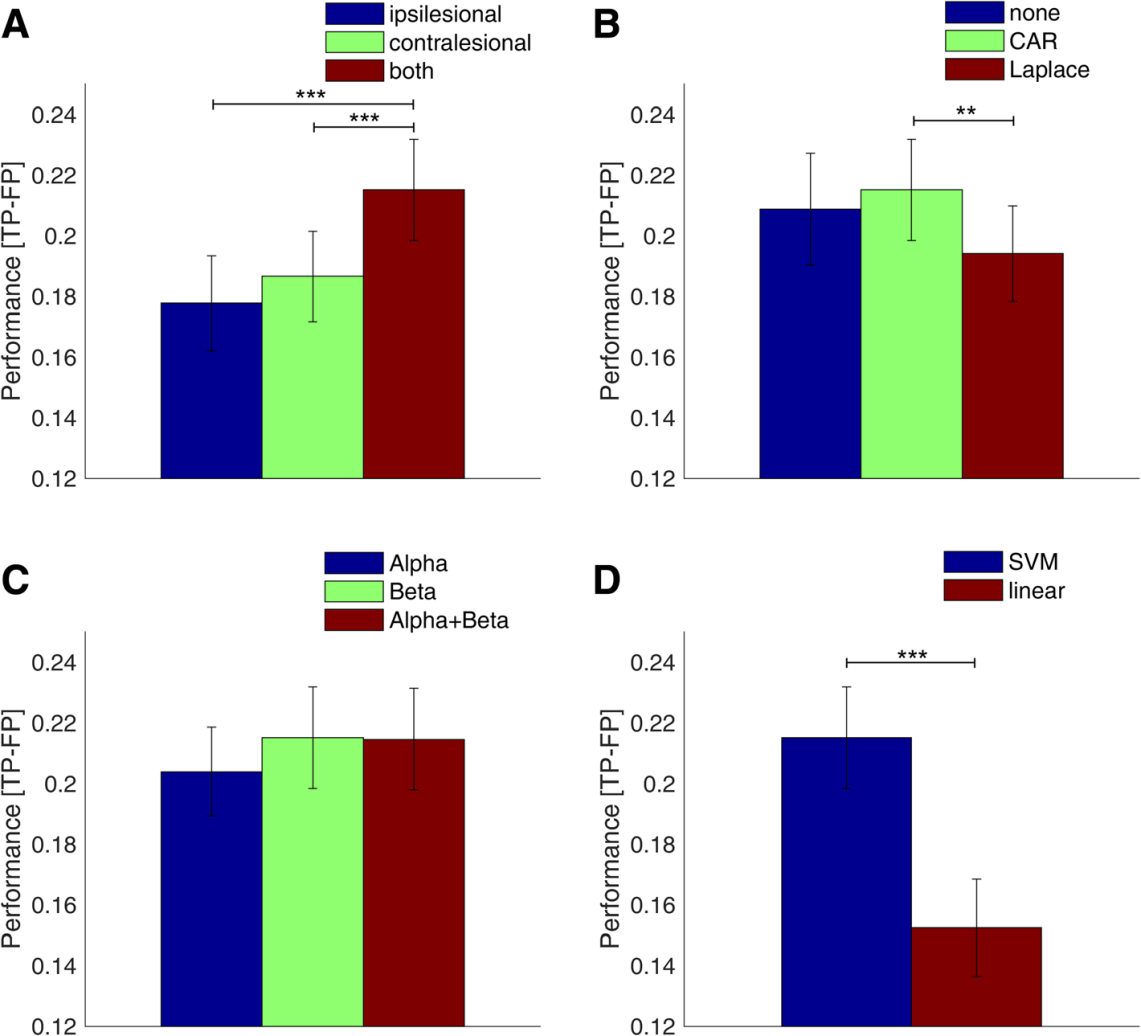
Classifier parameter effects on movement intention decoding accuracy. Average motor intention decoding accuracy (mean ± std) when using different configurations in terms of electrode placement (**a**), spatial filter (**b**), frequency band (**c**), and classifier (**d**). For each graph, the significance of the difference between the parameter with the highest accuracy and the other parameters was assessed using Wilcoxons ranksum test. The asterisks denote significant difference: * (*p* < 0.05), ** (*p* < 0.01), *** (*p* < 0.001)

We performed a 5-way ANOVA, considering decoding accuracy as dependent variable and patient, electrodes, frequency band, spatial filter, and classifier as factors. All the factors had a significant influence on accuracy (*p* < 0.0001). Overall, the use of electrodes from both hemispheres, CAR as spatial filter, the beta band, and the adaptive SVM classifier resulted in the best BMI accuracy. Based on this parameter combination, Fig. 2 shows the effects if each parameter is varied individually—maintaining the rest of the parameters constant. Regarding the electrode location (see Fig. 2a), the use of electrodes placed over both hemispheres was significantly superior (*p* < 0.001) than using electrodes placed over only one hemisphere, either ipsi- or contralesional. There was no significant difference between ipsi- or contralesional electrodes (*p* > 0.05). With respect to the spatial filter (see Fig. 2b), CAR had the highest decoding accuracy, which was significantly better than Laplacian spatial filtering (*p* < 0.01), but not significantly better than no spatial filtering (p > 0.05). For the choice of frequency bands (see Fig. 2c), beta had on average the highest decoding accuracy but the difference with the other frequency bands was not significant (p > 0.05). Regarding the classifier (see Fig. 2d), SVM yielded significantly higher decoding accuracy than the simple linear classifier used in the BMI intervention with patients (*p* < 0.001; Wilcoxon rank-sum test).

### Muscle vs brain decoding results

**Fig. 3:**
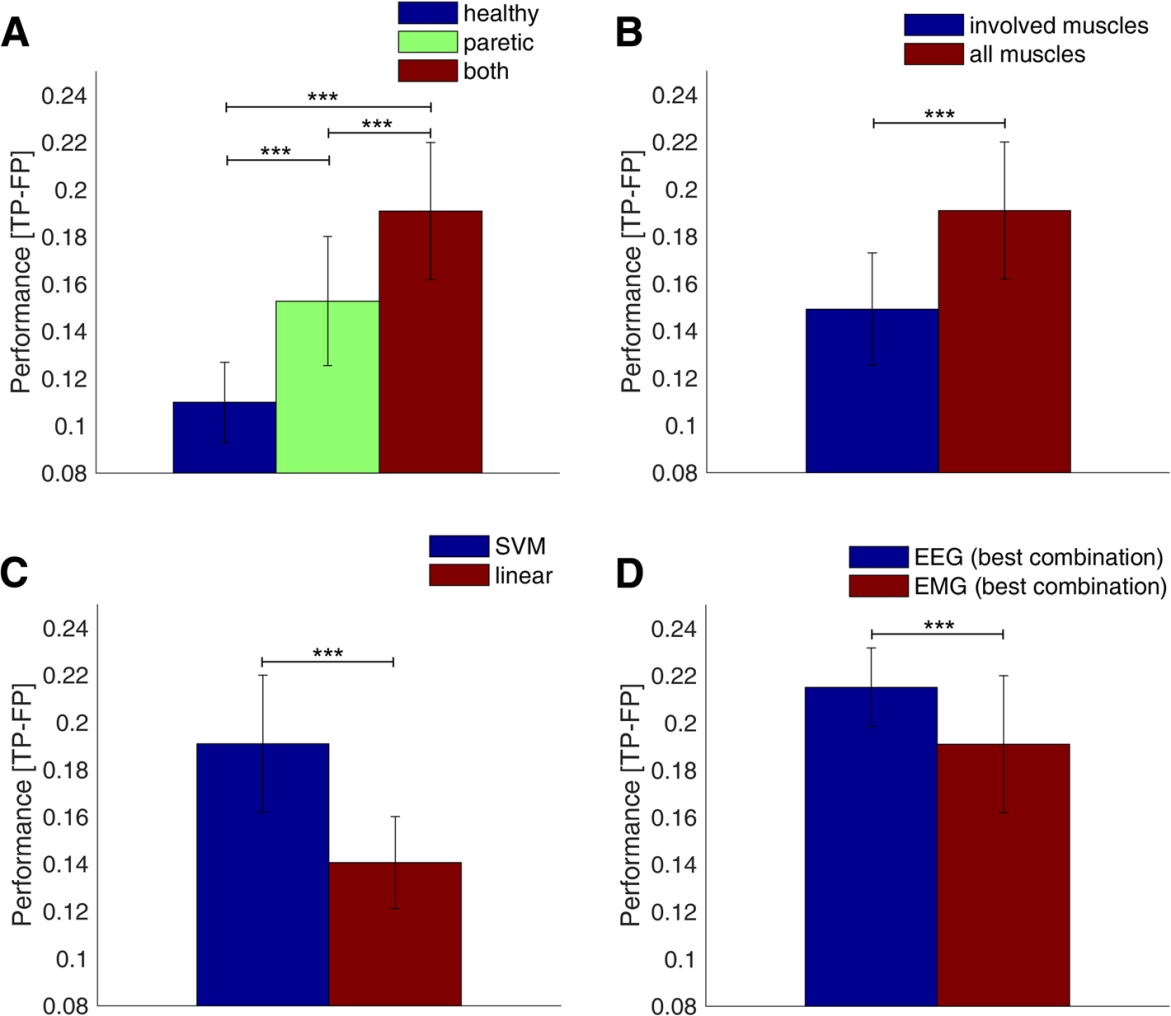
EMG movement intention decoding accuracy (mean ± std); **a**: Average accuracy using either EMG electrodes from healthy, paretic or both arms. **b**: Accuracy using different EMG electrodes. **c**: EMG classification accuracy using different classifiers. **d**: Comparison of classification accuracy using EEG and EMG signals. For each subplot, the significance of the difference between the parameter with the highest accuracy and the other parameters was assessed using Wilcoxons ranksum test. The asterisks denote significant difference: * (*p* < 0.05), ** (*p* < 0.01), *** (*p* < 0.001)

To compare the decoding accuracy using either brain signals (i.e., EEG) or muscle signals (i.e., EMG), we first evaluated the optimal parameters for EMG decoding. The use of the EMG data from all the electrodes placed on both sides and the SVM classifier yielded the best decoding accuracy (Fig. 3). Although using the EMG electrodes on the healthy arm only resulted in significantly worse decoding accuracy than using the electrodes on the paretic side (*p* < 0.0001), activity from both sides resulted in significantly better decoding accuracy than using electrodes only on one side (*p* < 0.0001). Decoding of healthy arm EMG activity demonstrates the presence of involuntary EMG activity that could affect EEG activity used for BMI control. A quantification of the potential bias of the BMI system caused by involuntary healthy upper-limb muscle activity is needed to determine how to remove, avoid or utilize the effect [37]. The use of all the EMG electrodes placed over the entire upper-limb (forearm and upper arm) resulted in a significantly higher decoding accuracy (*p* < 0.0001) than the use of the electrodes over muscles involved in the task only. We observed that SVM provided significantly better results for EMG too (*p* < 0.0001). A comparison of EEG and EMG decoding accuracy using the optimal parameter combination for both, shows that EEG allowed for a significantly better decoding accuracy (*p* < 0.0001) in chronic severely paralyzed stroke patients (see Fig. 3d).

## Discussion

The use of brain machine interfaces (BMI) for stroke rehabilitation may constitute a family of treatments that help improving the quality of life of those patients with the most severe motor deficits [1, 2, 38, 39]. Several different studies have already demonstrated the feasibility of BMIs to improve motor function when compared with placebo interventions [1–7]. Due to the novelty of this technology, there are still no standards to guide the researchers on how to design their rehabilitative BMI systems, and therefore there is a large heterogeneity between approaches. In this study, we evaluated the influence on decoding performance of different parameters for the design of a BMI to decode movement attempts in chronic stroke patients with complete hand paralysis.

Our results demonstrate that the analyzed components of the BMI (namely, the brain region considered to extract the cortical signals, the spatial filter, the frequency band and the classifier) have a significant influence on the average accuracy achieved, which we measured as the difference between true positives and false positives. The BMI design that showed the highest accuracy was using beta activity from both hemispheres, with a CAR filter, and an adaptive SVM classifier. In addition, for these patients, EEG activity also resulted to be superior to EMG.

The combination of activity from both cortical hemispheres provided significantly higher BMI performances than using the activity of each hemisphere alone, as expected from our previous findings [15, 16]. The EEG activity is affected after a stroke, and the degree of activation in the ipsilesional hemisphere measured during attempts of movements is smaller than in healthy population [40, 41]. This could explain why bihemispheric activity yielded better decoding results than using ipsilesional activity. However, contralesional activity only did not result in better decoding results than ipsilesional activity only, which might be reflecting a compensatory mechanism with complementary contralesional information to the one occurring in the ipsilesional area.

Nevertheless, it remains open whether contralesional or bihemispheric electrode placement would induce superior or inferior functional and cortical rehabilitation, and this analysis is out of the scope of this paper, as a new clinical trial using contralesional or bihemispheric activity during the same intervention would be needed. In our double-blind clinical study, we used ipsilesional activity to control robotic orthoses to enable the patients to move their paralyzed arm [2]. A recent study also proposed reinforcing ipsilesional activity during motor imagery with visual feedback, showing higher improvements in the patients than if no feedback is provided [6]. Other studies presenting rehabilitative BMIs for stroke combined the activity from both hemispheres to provide the feedback, although the clinical improvements in the patients reported were not significantly superior that the ones in the control interventions [3–5]. More recent works have also shown that contralesional activity can be used for BMIs [15, 42], although there are still no controlled trials proving the efficacy of this approach. To date, the role of the contralesional hemisphere for stroke motor rehabilitation is still not fully understood. Therefore, answering the question of what electrode placement is the best for motor recovery would require a randomized controlled clinical trial, fixing the type of EEG features and classifier, but varying the channels used to provide feedback in three patient groups (i.e., ipsilesional, contralesional, bihemispheric), or a much grounded theoretical hypothesis based probably on animal work [43].

The spatial filter that provided the best results was the CAR. Since no artifact removal method was used to resemble a realistic online scenario, it is plausible that some head movement, eye movements (electrooculography) or EMG related artefacts could have influenced the CAR filtering or the results with no filter at all, yielding in better decoding results [44]. Artifacts due to compensatory activity can be generated by (or correlated with) the attempts of movement of the patients, being learnt by the classifier and causing an optimistic bias that increases the BMI performance [44]. This is the reason why the Laplacian filter was used for the actual BMI intervention [2]. To avoid possible biases and guarantee that the BMI link is between the brain oscillations during movement intention and the actual movement, we recommend Laplacian filtering for online feedback, despite providing lower performance. Furthermore, CAR is a constant global spatial filter, and thus it does not help to capture the local spatial distributions of EEG components such as SMR, which we hypothesize is key to train and promote recovery. Although more sophisticated spatial filters have been shown to improve EEG decoding [45, 46], these methods need a higher amount of training data. This results in the need of additional calibration each session, prolonging the session time, which results in boredom and sleepiness with deterioration of performance. Complex spatial filters such as common spatial patterns (CSP) need a larger number of EEG electrodes to work properly. As time for BMI training is limited, time should be reserved for neuro-rehabilitation and keep the EEG preparation time and the BMI calibration to a minimum [47].

Concerning EEG oscillations, our results suggest that beta frequency is more adequate (although not significant) than alpha (mu). Furthermore, the individual SMR selected from each patient using the screening session and applied in the contingent group only during our BMI intervention was found to be sometimes in the mu and in other patients in the beta frequency range. Patients were trained to control their individual SMR (depending on feedback contingency) resulting in significant paretic limb movement intention decoding changes specific to the frequency band used online only. Therefore, it is plausible that the entrainment of individual oscillations (sometimes alpha, sometimes beta) affected the paretic limb movement intention decoding accuracy results when averaging and could explain why we did not obtain significant differences when using mu, beta or a combination of mu and beta as parameter for the decoding. Another plausible explanation for this finding might be that beta oscillatory activity, although affected by stroke [48], represents proprioceptive afferent activity [49], multisensory interactions during feedback [50] and has also been related to top-down attention and sensorimotor decision making [51]. Furthermore, as beta band is strengthened by the proprioceptive and haptic feedback it might result in better decoding performance (bias due to proprioceptive feedback), but it might not be a suitable feature to represent top-down motor volitional control. If one expects the operant conditioning effect to be significant, the best results should have been obtained using the online parameters, which was not the case (this can obviously only be checked in the experimental and contingent negative groups). However, we know that some parts of the BMI design were affected by the stroke (e.g. ipsilesional SMR modulation and SNR) and therefore BMI control was sometimes poor. The brain engages many networks to control and improve motor control (i.e., BMI control), and motor learning could significantly affect these neural networks producing indirect significant changes on them (e.g. oscillatory activity). Since no significant differences were found with regard to which frequency band produced better decoding performance, we suggest that one plausible approach for future BMI designs could be to have a predefined and wide frequency range (e.g., 7–30 Hz), and to use an automatic algorithm to identify the most reactive frequencies during movement attempts [52].

There is a large variety of classifiers that have been employed in the context of BMI [17, 18]. The choice of the classifier can have a significant impact on the BMI performance, although there is evidence suggesting that the features extracted to characterize the brain states to be classified might have a higher relevance than the classifier itself [19]. In BMI interventions requiring several sessions, adaptation or recalibration of the classifiers can be important to deal with the inherent non-stationarities of the EEG [47]. Our results showed how an adaptive SVM classifier outperformed the simple linear classifier that was used during the actual intervention with the patients. This suggests that future BMI rehabilitative interventions with stroke patients might benefit from the use of this type of classifier, although it should still be demonstrated if the improvement in performance would also entail higher motor recovery in the patients. Further research could also be conducted to evaluate other classification algorithms and measure how they affect BMI performance and subsequent recovery.

The results of BMI performance, measured as TP-FP, were generally around 0.2. For a non-biased classifier, this would correspond to values of around 60% of TP and 40% of FP. Although it may seem a low accuracy, notice that these values correspond to a continuous (i.e., pseudo-online) decoding, and not to the detection of single trials or onsets of movement. These values, although slightly lower, are not very dissimilar to values previously reported with healthy subjects [53]. This is in line with our previous results showing that the movement executions with the healthy limb of stroke patients can be decoded with higher accuracies than the attempts of movements with the paretic limb [16, 44], probably due to the low SNR of the activity of the ipsilesional cortex. It is important to note that most of the BMI clinical studies conducted with stroke patients do not report values about the performance of the BMI, which hinders comparisons. Recent works have proposed the classification of different types of movements with EEG only (e.g., different reaching directions [54] or different grasping types [55]), although the applicability of these approaches has not been demonstrated in online applications with patients.

Our results comparing EMG and EEG decoding emphasize the usefulness of an EEG-based BMI to learn the association between brain activity and movements. EMG, even if considering the best decoding accuracy offline, resulted in significantly worse outcome than EEG activity decoding. Although some patients present residual EMG activity that can be decoded with acceptable accuracy during several movements [24], EEG data yields superior movement intention decoding. We observed better EMG-based movement intention decoding in only 7 of 37 patients (patient IDs 2, 8, 10, 24, 32, 36, 27). Five of these patients had cFMA values higher than 10 (i.e., less impairment; patients 2, 24, 32, 36, 37), meaning more residual EMG activity. However, the other two had low cFMA values below 10, meaning that, despite not having much residual EMG activity, the decoding based on EEG was lower, probably due to a poor brain activation during the movement attempts [16]. In less handicapped stroke survivors, EMG decoding should result in equal or superior accuracy than EEG. However, the majority of patients with no or minimal residual movement do not show normal EMG amplitudes and may benefit better from EEG-based or even hybrid EEG-EMG interventions [22]. Nevertheless, if we use ipsilesional activity only, the EEG decoding might be poor compared to EMG motion intention decoding, and they might be representing different processes [56].

Operant conditioning depends on and directly affects every part of the BMI design, not only the BMI signal processing pipeline (electrode, spatial filter, frequency, EEG features to be used and classifier) but also the experimental protocol and feedback modality (motor task, visual, auditory and haptic feedback) and this might be a confounding factor of the here presented results. As an online testing of all these factors is not practical and the brain oscillatory activity used for the BMI depends on multiple neural networks directly and indirectly influenced by the operant conditioning, we consider the brain activity variability significantly larger than the one due to BMI design and therefore assume the above-mentioned confounding effects to be negligible. In this manuscript we limit our analyses to improve BMI performance and thus BMI control and we do not analyze the operant conditioning effect or BMI learning as we use all sessions of each patient to compute mean performance results and these effects might be even out in this analysis.

Note that all our analyses were conducted offline, simulating the online use of a BMI, on a dataset recorded during an actual closed-loop BMI intervention. Therefore, there are some factors that cannot be obviated and that might have an influence on our results. Firstly, the patients received proprioceptive stimulation by means of robotic orthoses that mobilized the arm/hand, and this generates afferent volleys that can modify the EEG activity [49]. Secondly, the patients were divided in three groups and received different types of association between the brain activity and the movement of the orthoses (i.e., contingent positive, contingent negative and sham). We believe that both of these factors do not have a big influence on our main conclusions, since all the analyses were equally applied to all the subjects. Note that the intervention group factor had an influence on performance when including the contingency as a factor in the ANOVA (*p* < 0.00001). Post-hoc analyses revealed a significant difference between experimental and contingent negative group (C+ vs C-; *p* < 0.00001) and between sham and contingent negative group (C- vs sham; *p* < 0.00001), while there was no significant difference between experimental and sham groups. The experimental and sham group resulted in similar BMI performance, while the performance in the contingent negative group (C-) was significantly lower than C+ or sham. This is reasonable, as they were rewarded (although not instructed) with exactly the opposite oscillatory changes that were trained in the control and faked in the sham groups. Although intervention group has an effect on performance, this factor does not influence our conclusion regarding what electrodes/spatial filter/frequency/classifier are optimal, because in the ANOVA the subject was modeled as one factor. As one subject only belonged to one group, the factor group is implicitly modelled in this case and therefore the factor group does not influence the results regarding what methods are optimal and their statistical significance.

None of the patients in the sham group reported any confusion or any perception of inconsistency throughout the whole treatment. Placebo effects were tested and did not result in significance difference between patients groups [2]. Finally, it is important to note that the conclusions extracted from our analyses can only be applied to the population of patients studied: i.e., chronic stroke with complete hand paralysis. However, stroke patients with different degree of affection might obtain different results, and further research should be conducted to extend our results to other typologies of patients.

## Conclusions

This work validated different methodologies to design decoders of movement intentions for completely paralyzed stroke patients. Using a clinically-relevant dataset of 37 patients, we provide strong evidence of the relevance of different parameters for designing clinical brain machine interfaces (BMI). The design with the highest performance was found to be the use of bihemispheric beta activity, applying a common average reference (CAR) and an adaptive SVM classifier. The EMG activity provided significantly lower decoding results than the EMG activity to identify movement intention. We proposed several methodological recommendations to optimize SMR-based BMI performance for stroke patients. Further investigation should be conducted to evaluate to what extent the approaches leading to higher accuracies also induce higher motor recovery in paralyzed stroke patients.

## Abbreviations

ANOVA: Analysis of variance
BMI: Brain-machine interface
CAR: Common average reference
EEG: Electroencephalography
EMG: Electromyography
ERD/ERS: Event-related desynchronization/synchronization
FP: False positive
SMR: Sensorimotor rhythm
SVM: Support vector machine
TN: True negative
TP: True positive
WL: Waveform length
